# Receptor model-based sources and risks appraisal of potentially toxic elements in the urban soils of Bangladesh

**DOI:** 10.1016/j.toxrep.2023.02.011

**Published:** 2023-02-24

**Authors:** Tapos Kumar Chakraborty, Md Shahnul Islam, Gopal Chandra Ghosh, Prianka Ghosh, Samina Zaman, Md Ripon Hossain, Ahsan Habib, Md Simoon Nice, Md Sozibur Rahman, Khandakar Rashedul Islam, Baytune Nahar Netema, Suvasish Das Shuvo, Nazmul Hossain, Abu Shamim Khan

**Affiliations:** a Department of Environmental Science and Technology, Jashore University of Science and Technology, Jashore 7408, Bangladesh; b Department of Nutrition and Food Technology, Jashore University of Science and Technology, Jashore 7408, Bangladesh; c Department of Computer Science and Engineering, Jashore University of Science and Technology, Jashore 7408, Bangladesh; d Environmental Laboratory, Asia Arsenic Network, Jashore 7400, Bangladesh

**Keywords:** Bangladesh, Ecological risk, Hazardous elements, Public health, Urban land use

## Abstract

Rapid urbanization and industrial development have prompted potentially toxic elements (PTEs) in urban soil in Bangladesh, which is a great concern for ecological and public health matters. The present study explored the receptor-based sources, probable human health and ecological risks of PTEs (As, Cd, Pb, Cr, Ni, and Cu) in the urban soil of the Jashore district, Bangladesh. The USEPA modified method 3050B and atomic absorption spectrophotometers were used to digest and evaluate the PTEs concentration in 71 soil samples collected from eleven different land use areas, respectively. The concentration ranges of As, Cd, Pb, Cr, Ni, and Cu in the studied soils were 1.8—18.09, 0.1—3.58, 0.4—113.26, 0.9—72.09, 2.1—68.23, and 3.82—212.57 mg/kg, respectively. The contamination factor (CF), pollution load index (PLI), and enrichment factor (EF) were applied to evaluate the ecological risk posed by PTEs in soils. Soil quality evaluation indices showed that Cd was a great contributor to soil pollution. The PLI values range was 0.48–2.82, indicating base levels to continuous soil degradation. The positive matrix factorization (PMF) model showed that As (50.3 %), Cd (38.8 %), Cu (64.7 %), Pb (81.8 %) and Ni (47.2 %) were derived from industrial sources and mixed anthropogenic sources, while Cr (78.1 %) from natural sources. The highest contamination was found in the metal workshop, followed by the industrial area, and brick filed site. Soil from all land use types revealed moderate to high ecological risk after evaluating probable ecological risks, and the descending order of single metal potential ecological risk was Cd > As > Pb > Cu > Ni > Cr. Ingestion was the primary route of exposure to potentially toxic elements for both adults and children from the study area soil. The overall non-cancer risk to human health is caused by PTEs for children (HI=0.65 ± 0.1) and adults (HI=0.09 ± 0.03) under USEPA safe limit (HI>1), while the cancer risks from exclusively ingesting As through soil were 2.10E-03 and 2.74E-04 for children and adults, respectively, exceeding the USEPA acceptable standard (>1E-04).

## Graphical Abstract

**Figure:**
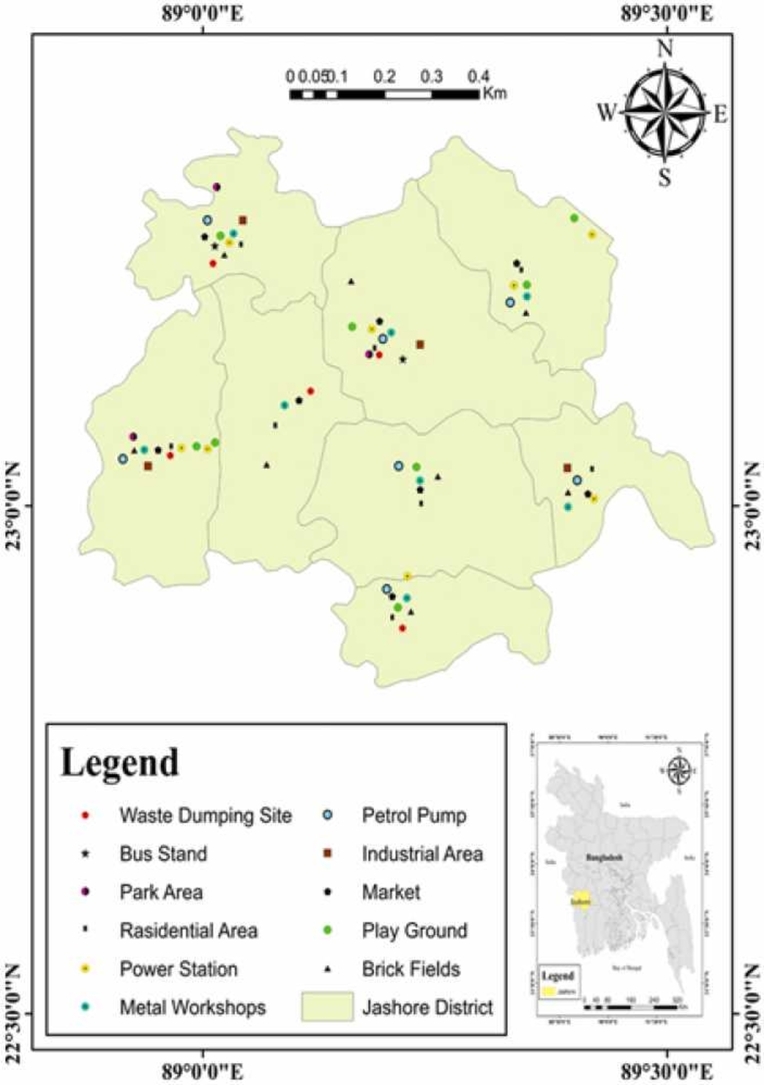

## 1 Introduction

Soil pollution by PTEs is a matter of great concern because of their source abundance, richness, slow breakdown, higher accumulating rate, and probable eco-toxic nature for humans and other biota [1], [2], [3]. Soil is a valuable natural reserve for the existence of biotic components [2], [4], which is being contaminated by PTEs from manmade activities, including waste disposal, sewage effluent, agricultural practices, industrial activities, vehicular exhaust, etc [5]. In comparison to water and air, soil can acquire larger amounts of organic and inorganic substances because of their diverse influencing factors and increasing area [6]. Nowadays, soil pollution by PTEs in urban areas is a highly remarkable environmental issue for developed and developing countries [7]. In urban areas, soil pollution is gradually increasing because of rapid urbanization and industrialization activities [8]. This problem is common in Bangladesh because many landowners switch their agricultural land for diverse purposes, such as shopping malls, markets, workshops, storage sites, etc., for high economic benefits [9]. The distribution of toxic metals in urban area soil varies from place to place due to the characteristics of parent materials, climate, soil morphology, and anthropogenic interferences [10], [11]. Therefore, urban soil act as a sink of pollution due to the continuous receiving of an excessive amount of PTEs, consequently leading to the changing in soil chemistry, soil ecology, and soil functioning capacity [12], [13], [14]. The studies of probable ecological risk evaluation and Source attribution of PTEs in contaminated soil have attained more attention [15]. Diverse environmental risk evaluation approaches apply when data is taken from non-polluted areas [16], [8], [2], [17], [18]. Soil serves as a range of habitats, stores massive contaminants, and significantly contributes to environmental and geochemical speciation, so surface soil sampling is a fast, inexpensive, effective, and significant information-carrying system for evaluating the quality of terrestrial ecosystems [19], [20]. Conversely, the bioavailability of toxic metals in urban soil can pose substantial human health risks if they entered the human body via dermal contact, incidental ingestion, and inhalation [21], [22], [23]. The PTEs exposure level and potential health risk depend on different toxicity influencing factors, such as chemical type, age, gender, dose-time relation, mode of exposure, metabolic rate and distribution within the body of the host etc. [24]. Children and infants are more vulnerable to PTEs exposure and potential health risk than adults because they may inhale, ingest and dermally absorb greater amounts of heavy metals by involving their hands, mouth, or even the whole body during their outdoor and play activities in the urban environment [25], [26], [27]. After exposure, PTEs are stored in the human body (eg. bloodstream, gastrointestinal and intestinal membranes) that either create heavy metal-associated health hazards or act as a co-factor of several diseases, while older people are highly threatened due to their lower disease defence capacity [28], [3], [29]. So, to determine and define the qualitative (cancer-causing and non-cancer-causing health concerns) and quantitative (mapping and calculation) risk levels, methods for risk assessment approach are required [26], [17]. Moreover, the long-period accumulation of PTEs not only degrades the soil quality and their adjacent environment but also poses a significant adverse effect on human health. Therefore, it is essential to investigate the distribution of PTEs and the identification of sources in different types of urban soil usage to evaluate the level of contamination and develop remediation strategies. Conversely, detailed source dispersal of PTEs in urban land use soil is far away from scientific consideration yet this study is vital for public health concerns. Due to the variety and complexity of the pollution sources, using a single scientific method to draw pollution zones is challenging. To identify the specific and acceptable results of PTEs sources, this study applied principle component analysis (PCA), and receptor model (e.g. PMF). This study's endeavour to incorporate different receptor models (PCA, and PMF) may be an appropriate strategy for the different pollution sources scattered throughout Bangladesh urban soils. The Jashore district was selected for this study as the location that has drawn the most attention owing to experiencing a variety of environmental issues as a result of the fast expansion of infrastructure and industrial activity. Monitoring the distribution of poisonous metal levels in urban soil surfaces is therefore required to compare with non-polluted background values and evaluate the environmental health hazard and ecological sustainability for this area. Though several studies have been conducted in agricultural soils of Bangladesh for determining the metal concentration such as industrial areas in Dhaka [30], Dhaka Export Processing Zone [31], Dhaka Export Processing Zone Area [32], Patuakhali district [5], industries area of Dhaka city [33], the evidence on PTEs in the urban soil by land-use changes in Jashore district especially using PMF model is inadequate. Therefore, the objectives of this study aimed to identify (1) the concentrations of PTEs (As, Cd, Pb, Cr, Ni, and Cu) in different land-use urban soils; (2) to find out the potential sources of PTEs in the urban soil using receptor model-based sources; and (3) to analyze the possible risks of PTEs to ecological and human health.

## 2 Materials and methods

### 2.1 The study area

**Fig. 1:**
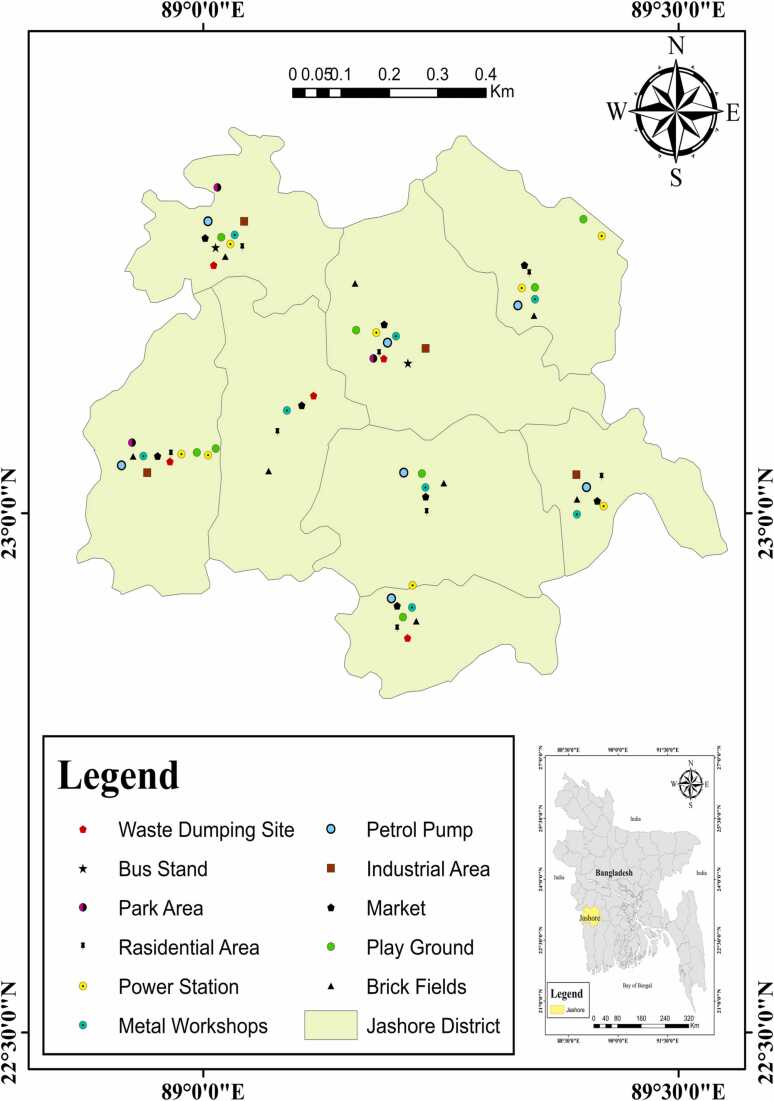
Map of the study area (Jashore district, Bangladesh).

Jashore is one of the more significant districts of the Khulna division, located in Bangladesh southwestern region. Geographically it is located at 22°10 ´ and 22°28 ´ east longitudes and between 89°16 ´ and 89°64 ´ north latitudes (Fig. 1). This study area was exposed to a variety of pollutants from the outflow of urban areas, heavy traffic, and airborne fallout, as well as solid waste and untreated sewage from numerous industrial sites (such as brickfields, chemical industries, tanneries, and, textile). The country's biggest Land port (Benapol) is situated in this area, mainly used for import and export trading between Bangladesh and India. Lithologically, this area is formed by the Ganges deltaic action, and it is also situated at Gopalganj-Khulna Bills agro-ecological zone no 14.

Between the Ganges River Floodplain as well as the Ganges Tidal Floodplain, the region comprises large low-lying regions, and clay loam soils are predominant in the Jashore district [34]. The yearly rainfall is 1537 millimetres (60.5 in.) and the yearly mean temperature ranges from 15.4° to 34.6°C (59.7–94.3 °F) [35], which significantly influences the concentration and dispersal of metal and metalloids in elution from both soil and surface runoffs [36]. Conversely, the whole area of the district is 2606.98 square km, 7 m elevated from sea level, with a population of a total of 2764,547 [37], and the main rivers are the Bhairab, Chitra, Betna, Kopotakkho, and the Mukteshwari, while this district consists of eight Upazila [38].

### 2.2 Collection, processing, and metal extraction of soil samples

This research was performed in 11 different land uses sites i.e., brickfield (BF), playground (PG), market (M), park area (PA), industrial area (IA), petrol pump (PP), metal workshop (MW), bus stand (BS), power station (PS), residential area (RA), and waste dumping area (WDA), where surface soil samples were collected during the January to March 2021 from 71 sampling points (Fig. 1), at a depth of 0–15 cm using a stainless-steel scoop. Three subsoil samples were taken at each location following the standard protocol [39]. These sub-soil samples were appropriately mixed to make a composite sample. The study soil sample was taken from these composites and stored in nonmetallic sample collecting bags to avoid unwanted contaminations. The obtained samples were dried for 24 h at 80 °C in the oven (Labtech LDO-150 F, Korea) then crushed using a crusher and sieved (2 mm) [40]. For lab analysis, the samples were then placed in Pyrex-sealed glass vials. The pre-industrial soil sample for background value was collected and prepared by following the procedures of Islam et al., 2015. Analytical grade chemicals (Merck, Germany) were used throughout the whole analysis. Modified USEPA Method 3050B [41] was used to carry out the entire soil digestion to determine the presence of toxic metals. To summarize, 2.0 g of every soil sample was sequentially digested utilizing concentrated 10 mL HNO_3_ (69 %, Merck, Germany; 95^0^ C for 2 h), 5 mL H_2_SO_4_ (98 %, Merck, Germany; 250^0^ C for 2 h), 5 mL HClO_4_ (70 %, Merck, Germany; 300^0^ C for 2 h) and 3 mL H_2_O_2_ (30 %, Merck, Germany; 300^0^ C for 30 min). Following digestion, solutions were extracted using Whatman no. 41 filter paper (pre-washed with 0.1 M HNO_3_), and the final volume was created using 100 mL of double-distilled water.

### 2.3 Chemical investigation and quality control

The physicochemical composition of soil samples' such as electrical conductivity (EC) (HACH Sension −156; multi-parameter, USA), soil texture (sand, silt, and clay), pH meter (Model: MARTINI instruments, pH 56 pHWP, USA), percentage of organic carbon (OC), and percentage of organic matter (OM) was determined using standard procedure, adopted from Kormoker et al. [17]. A hydride Generator, graphite furnace and air-acetylene flame incorporated Atomic-absorption-spectrophotometer (AAS) (Model: AA-7000, SHIMADZU, Japan), was used to measure the concentration of Cd, As, Pb, Cr, Cu, Ni, and Fe in the samples with single element hollow cathode lamp operating at the wavelength of 228.80, 193.70, 283.3, 357.90, 324.80, 232.00 and, 248.30 nm, respectively. The detection limit of Cd and As was 0.004 and 0.0003 mg/kg, respectively, whereas, for Cr, Cu, Fe, Ni, and Pb was 0.013–0.070 mg/kg. About 98.5 % purity and 1.82 L/min average flow rate of acetylene were used for sample analysis. The standard solution (1000 ppm) from Sigma Aldrich, Switzerland, was used for instrument calibration. The results were expressed as mg/kg for soil samples. Throughout the experiment, double-distilled water was used. Glassware and other laboratory supplies were utilized after being thoroughly cleaned and dried. For quality control, every sample, both verified and blank was made to run. A certified reference material (CRM) for heavy metals entitled DORM-4 Fish Protein was used to test the analytical process. The Canadian National Research Council (NRC) provided the CRM. The recoveries revealed a substantial relationship between certified and observed values and were within 88.15–112.5 % (Table S1) of the certified values.

### 2.4 Appraising the contamination level of PTEs

Different pollution evaluation indices, including the enrichment factor (*EF*), geo-accumulation index (*I*_*geo*_), contamination factor (*CF*_*i*_), degree of contamination (*C*_*d*_), pollution load index (*PLI*), and, potential ecological risk evaluation index (*PERI*) were used in this study to assess the degree of pollution, details presented in Table 2 and pollution/risk evaluation category are given in Table S2.

**Table 1:** Pollution evaluation indices.

Evaluation indices	Equation	Explanation	Reference
Enrichment factor (*EF*)	$\mathrm{EF}=\frac{{\left(\frac{C_{x}}{M_{\mathrm{Fe}}}\right)}_{\mathrm{sample}}}{{\left(\frac{C_{x}}{M_{\mathrm{Fe}}}\right)}_{\mathrm{background}}}$ (1)	(*C*_*x*_*/ M*_*Fe*_) sample = the ratio of the concentration of toxic metals (*C*_*x*_) to that of iron (*M*_*Fe*_) in the soil sample (*C*_*M*_*/C*_*Fe*_) background = the same reference ratio in the pre-industrial sample	Chakraborty et al. [42], Islam et al. [7], Sutherland [43]
Geo-accumulation index (*I*_*geo*_)	$I_{\mathrm{geo}}={\mathrm{Log}}_{2}\left(\frac{C_{\mathrm{xi}}}{{1.5\times B}_{\mathrm{xi}}}\right)$ (2)	*C*_*xi*_ = the studied toxic metal concentration of the i^th^ parameter *B*_*xi*_ = the geochemical background value of the toxic metal of the i^th^ parameter in the preindustrial soil samples of the study area	Muller [44], Islam et al. [45]
Contamination factor (*CF*_*i*_)	${\mathrm{CF}}_{i}=\frac{C_{\mathrm{xi}}}{B_{\mathrm{xi}}}$ (3)		Hakanson [46]
Degree of contamination (*C*_*d*_)	$C_{d}=\sum_{i=1}^{n}{\mathrm{CF}}_{i}$ (4)	*C*_*d*_ = the degree of contamination	Hakanson [46]
The pollution load index (PLI)	$\mathrm{PLI}=\sqrt[{n}]{{\mathrm{CF}}_{i1}\times {\mathrm{CF}}_{i2}\times {\mathrm{CF}}_{i3}\ldots \ldots \ldots \ldots \times {\mathrm{CF}}_{\mathrm{in}}}$(5)	n = number of studied metals	Tomlinson et al. [47]
Potential ecological risk evaluation index (*PERI*)	$E_{r}^{i}=T_{r}^{i}\times {\mathrm{CF}}_{i},\mathrm{PERI}=\sum_{i=1}^{n}E_{r}^{i}$ (6)	*E*_*r*_^*i*^ = the particular metal-induced ecological risk index of the i^th^ parameter *T*_*r*_^*i*^ = the biological toxic factor of the i^th^ parameter The study used the *T*_*r*_^*i*^ value of As, Ni, Pb, Cd, Cr, and Cu as 10, 6, 5, 30, 2, and 5, respectively	Hakanson [46]

**Table 2:** Human health risks assessment methods via ingestion, dermal contact and inhalation exposure routes.

Assessment approaches	Equation
Chronic daily intake (*CDI*) (mg/kg/day)	${\mathrm{CDI}}_{\mathrm{ingest}}=\frac{\mathrm{CS}\times \mathrm{IRS}\times \mathrm{EF}\times \mathrm{ED}}{\mathrm{BW}\times \mathrm{AT}}\times \mathrm{CF}(7){\mathrm{CDI}}_{\mathrm{dermal}}=\frac{\mathrm{CS}\times \mathrm{SA}\times \mathrm{AF}\times \mathrm{ABS}\times \mathrm{EF}\times \mathrm{ED}}{\mathrm{BW}\times \mathrm{AT}}\times \mathrm{CF}(8){\mathrm{CDI}}_{\mathrm{inhale}}=\frac{\mathrm{CS}\times \mathrm{InhR}\times \mathrm{EF}\times \mathrm{ED}}{\mathrm{PEF}\times \mathrm{BW}\times \mathrm{AT}}$ (9)
Hazard quotient (*HQ*)	${\mathrm{HQ}}_{\mathrm{ingest}}=\frac{{\mathrm{CDI}}_{\mathrm{ingest}}}{\mathrm{RfD}}$ (10) ${\mathrm{HQ}}_{\mathrm{dermal}}=\frac{{\mathrm{CDI}}_{\mathrm{dermal}}}{\mathrm{RfD}}(11){\mathrm{HQ}}_{\mathrm{inhale}}=\frac{{\mathrm{CDI}}_{\mathrm{inhale}}}{\mathrm{RfD}}(12)$
Hazard index (*HI*)	$\mathrm{HI}=\sum \mathrm{HQ}={\mathrm{HQ}}_{\mathrm{ingest}}+{\mathrm{HQ}}_{\mathrm{dermal}}+{\mathrm{HQ}}_{\mathrm{inhale}}(13)$
Carcinogenic risk (*CR*)	${\mathrm{CR}}_{\mathrm{ingest}}={\mathrm{CDI}}_{\mathrm{ingest}}\times {\mathrm{CSF}}_{i}$ (14) ${\mathrm{CR}}_{\mathrm{dermal}}={\mathrm{CDI}}_{\mathrm{dermal}}\times {\mathrm{CSF}}_{i}$ (15) ${\mathrm{CR}}_{\mathrm{inhale}}={\mathrm{CDI}}_{\mathrm{inhale}}\times {\mathrm{CSF}}_{i}$ (16) $\mathrm{Lifetime cancer risk}={\mathrm{CR}}_{\mathrm{ingest}}+{\mathrm{CR}}_{\mathrm{dermal}}+{\mathrm{CR}}_{\mathrm{inhale}}$ (17)

### 2.5 Human health risk assessment

This study estimates the human health risks (non-carcinogenic and carcinogenic) due to exposure to contaminated soil via ingestion, dermal contact, and inhalation exposure pathways for children and adults. USEPA [48], [39] proposed health risk assessment method was applied (Table 2), and model parameters and references dose are presented in Table 3 and Table S3, respectively.

**Table 3:** Exposure parameters and their values were adopted from USEPA [49], [50].

Parameter	Unit	Child	Adults
Body weight (*BW*)	kg	15	70
Exposure frequency (*EF*)	days/year	350	350
Exposure duration (*ED*)	years	6	30
Ingestion rate (*IR*)	mg/day	200	100
Inhalation rate (*IRair*)	m^3^/day	10	20
Skin surface area (*SA*)	cm^2^	2100	5800
Soil adherence factor (*AF*)	mg/cm^2^	0.2	0.07
Dermal Absorption factor (*ABS*)	none	0.1	0.1
Dermal exposure ratio (*FE*)	none	0.61	0.61
Particulate emission factor (*PEF*)	m^3^/kg	1.3 × 10^9^	1.3 × 10^9^
Conversion factor (*CF*)	kg/mg	10^−6^	10^−6^
Average time (*AT*)			
For carcinogens	days	365 × 70	365 × 70
For non-carcinogens		365 × ED	365 × ED

### 2.6 Positive matrix factorization (PMF)

PMF (EPA PMF version 5.0) is a mathematical receptor model, used in this study to distribute the source of toxic metals in the urban soil. It implements well to calculate summary and source contribution according to reliable factorization algorithms; mathematically it can be stated as Eq. (18):

(18) $$X_{\mathrm{iy}}=\sum_{j=1}^{a}g_{\mathrm{ia}}f_{\mathrm{ay}}+e_{\mathrm{iy}}$$

Where, x_iy_ is the i^th^ species value calculated in the y^th^ sample; f_ay_ is the input of a^th^ source to y^th^ sample; g_ia_ is the value of i^th^ species from y^th^ source, and e_iy_ is the methodical error. The objective of this model was to discover the values for g_ia_ and f_ay_, which best repeat the measured value x_iy_. These data were perfected until the lowest Q value was attained, where Q is defined as Eq. (19).

(19) $$Q(A)=\sum_{y=1}^{m}\sum_{i=1}^{n}\left(\frac{e_{\mathrm{iy}}}{\alpha_{\mathrm{iy}}}\right)$$

Here the α_iy_ states the “uncertainty” in the i^th^ species of sample number y. heavy metals concentration and their agreeing uncertainties are the input data for the model, which are evaluated based on PMF fundamentals (version 5.0) and User Guide. The data below the detection limit (MDL) are replaced with the methods as follows in (20), (21).

(20) $$\alpha_{\mathrm{iy}}=\frac{5}{6}\times \mathrm{MDL}\left(X_{\mathrm{iy}}\leq \mathrm{MDL}\right)$$

(21) $$\alpha_{\mathrm{iy}}=\left(0.05\times X_{\mathrm{iy}}\right)+\mathrm{MDL}\left(X_{\mathrm{iy}}\geq \mathrm{MDL}\right)$$

Once the lower Q values, higher R^2^ values, and clear interpretability of the factors were attained, the data were run 20 times using this model with a random seed and a varied number of factors (varying from 3 to 6). All runs' Q (robust) and Q (True) values were well examined, and run 13 was chosen for factor extraction, with steps beginning at least 127. Positive matrix factorization model accuracy data are presented in Table S4.

### 2.7 Statistical analysis

Multivariate statistical techniques such as principal component analysis (PCA), cluster analysis (CA), and Pearson's correlation matrix (PCM) were used to determine the most likely sources of toxic harmful metals in the soil. To explain the study findings, PCA was performed using the mean value of the individual variable, whereas each principal component's loading value > 0.5 and eigenvalue > 1 were taken from the analysis table. The Ward technique, Euclidean distance, and standardized data sets (Z-cores) were employed for CA. The dendrogram, which is graphically displayed, demonstrates how each examined toxic metal is connected to a cluster. This study analyzed data using Microsoft Excel-2010, SPSS (V.20), and ArcGIS (V.10.5) software.

## 3 Results and discussion

### 3.1 Physiochemical characteristics and concentration of PTEs in soil samples

The soil pH values varied from 7.15 to 8.36 (Table S5), indicating that the slightly alkaline might be the decaying of organic matter as well as discharging of wastes, effluents, chemicals and salt etc in soil [51]. Soil Resources Development Institute (SRDI), Bangladesh, suggested that this study area's soils are in the non-saline to low saline category. The percentage of organic matter ranged from 1.09 to 5.65% (Table S3), due to the deposition or decomposition of the larger amount of waste and sewage sludge. Several studies recommended that polluted soil contains more organic matter than non-polluted soil [51]. Based on the USDA soil texture classification, this study area's soil is sandy loam (Table S5). The PTEs concentration ranges in the soil samples of different land uses of the study areas were as follows: As (1.8—18.09 mg/kg); Pb (0.4—113.26 mg/kg); Cd (0.1—3.58 mg/kg); Ni (2.1—68.23 mg/kg); Cr (0.9—72.09 mg/kg); Cu (3.82—212.57 mg/kg) (Table 1.). Changes in the point and non-point sources of pollution may be responsible for the fluctuation of PTEs. The descending order of the mean PTEs concentration in soil samples was Cu > Cr > Pb > Ni > As > Cd (Table 4). The relatively high standard deviation for some metals (eg. Cr, Cu, and Pb) in different land uses exhibited relatively substantial spatial variability in these metals [52]. Potential toxic element concentrations in study locations followed the decreasing order of site MW > IA > BF > BS > WDA > M > PS > PP > PA > PG > RA (Table 4). In the soils of the MW site (Cu=124.67 ± 48.49 mg/kg), the highest amount of Cu was discovered, followed by the BS site (Cu=42.04 ± 24.38 mg/kg) due to the metal workshop and waste-burning activities. In terms of soil Cu content, around 77 % and 27 % of samples surpassed TRV and DSQS, respectively. At the M site (48.75 ± 16.52 mg/kg), a high amount of Cr was found, followed by the BS site (41.15 ± 19.11 mg/kg) due to the disposal of untreated waste material (eg. plating, anti-corrosion treatment by-products, etc. [53], [54]. For Cu, about 66 % and 19 % of samples exceeded TRV and ASQS, respectively. Higher concentrations of Pb were found in soil 78.36 ± 35.04 and 64.36 ± 27.69, mg/kg for IA and MW sites, respectively, due to the releasing of Pb-contaminated waste (eg. Lead-acid batteries, storage tanks, leaded gasoline, and paint, lead solders) from these sites. Considering the Pb concentration, about 30 % and 7 % of samples exceeded the TRV and CSQS, respectively (Table 4). At the MW site (51.52 ± 12.03 mg/kg), soil contains an elevated level of Ni, followed by the BF site (20.68 ± 7.54 mg/kg) due to additions or discharging of Ni-containing waste (eg. coal, crude oil, electroplating by product, etc.) from the brickfield, and metal workshop. Nearly 48 % of the samples in the study region exceeded TRV and 10 % exceeded DSQS for Ni. The maximum mean As concentration was assessed in the MW site soil (11.39 ± 4.23 mg/kg), and BF site (10.16 ± 3.27 mg/kg), where 59 % of samples exceeded TRV and 14 % of samples exceeded the CSQS value. Various metallic waste, burning of fossil fuels, emissions, and waste from brickfields and metal workshop areas are the main sources of As [55].

**Table 4:** The concentration of potentially toxic elements (mg/kg dw) in soils collected from Jashore district, Bangladesh.

Land use type		As	Cd	Pb	Cr	Ni	Cu
PA (N = 3)	Mean ± SD	8.56 ± 4.32	0.85 ± 0.18	6.78 ± 6.48	38.85 ± 18.27	12.99 ± 6.23	15.64 ± 7.03
	Range	3.32–13.9	0.7–1.05	1.16–13.87	20.09–56.58	5.98–17.88	9.13–23.1
PG (N = 8)	Mean ± SD	7.91 ± 3.61	0.78 ± 0.40	8.60 ± 4.19	30.44 ± 9.70	15.72 ± 6.31	16.79 ± 6.18
	Range	2.13–13.86	0.1–1.2	2.74–15.34	18.03–43.01	5.32–23.1	5.58–28.35
M (N = 8)	Mean ± SD	5.15 ± 2.31	0.98 ± 0.45	24.50 ± 10.44	48.75 ± 16.52	9.29 ± 4.18	32.14 ± 9.61
	Range	1.8–8.88	0.3–1.8	11.37–38.93	17.09–72.09	2.19–14.23	11.91–43.14
BF (N = 8)	Mean ± SD	10.16 ± 3.27	1.09 ± 0.61	28.87 ± 10.65	40.24 ± 14.52	20.68 ± 7.54	33.48 ± 8.70
	Range	4.6–14.5	0.23–2.1	13.79–45.76	12.01–59.09	7.18–29.11	14.51–44.1
IA (N = 4)	Mean ± SD	6.69 ± 3.01	1.21 ± 0.58	78.36 ± 35.04	29.51 ± 7.39	16.50 ± 5.78	19.25 ± 3.98
	Range	3.76–11.54	0.7–2.03	34.58–113.26	21.04–37.09	8.1–21.3	15.1–24.3
PP (N = 8)	Mean ± SD	6.81 ± 2.75	0.94 ± 0.31	15.48 ± 9.44	33.69 ± 10.58	10.05 ± 5.28	28.47 ± 14.03
	Range	2.45–11.21	0.3–1.23	5.66–28.7	19.77–50.08	2.1–18.88	10.24–52.15
MW (N = 8)	Mean ± SD	11.39 ± 4.23	2.05 ± 0.84	64.36 ± 27.69	33.45 ± 14.62	51.52 ± 12.03	124.67 ± 48.49
	Range	5.99–18.09	1.02–3.58	33.1–102.78	15.02–55.12	29.89–68.23	65.97–212.57
BS (N = 3)	Mean ± SD	6.96 ± 4.40	1.25 ± 0.86	27.90 ± 15.02	41.15 ± 19.11	14.27 ± 4.41	42.04 ± 24.38
	Range	2.13–12.78	0.4–2.11	13.68–43.6	24.55–62.04	9.19–17.13	21.82–69.12
PS (N = 8)	Mean ± SD	7.77 ± 3.90	0.94 ± 0.64	9.49 ± 6.29	37.88 ± 12.96	17.09 ± 6.98	26.18 ± 11.52
	Range	2.56–14.69	0.1–1.87	2.07–19.06	13.99–58.09	3.19–25.87	4.23–42.11
RA (N = 8)	Mean ± SD	5.67 ± 2.72	0.60 ± 0.35	4.09 ± 4.33	5.87 ± 4.73	10.17 ± 5.00	12.53 ± 7.89
	Range	1.9–10.7	0.1–1.03	0.4–12.09	0.9–12.09	2.98–18.23	3.82–28.3
WDA (N = 5)	Mean ± SD	6.27 ± 2.49	1.24 ± 0.56	29.75 ± 11.20	38.54 ± 13.47	19.00 ± 8.14	32.88 ± 10.87
	Range	3.66–10.87	0.69–2.01	12.09–41.01	18.03–55.08	11.94–32.12	22.89–51.32
BG^a^		4.90	0.30	20.00	41.00	22.00	27.00
DSQS^b^		29.00	0.8	85.00	100.00	35.00	36.00
CSQS^c^		12.00	1.4	70.00	64.00	50.00	63.00
TRV^d^		6.00	0.6	31.00	26.00	16.00	16.00
ASQS^e^		20.00	3.00	300.00	50.00	60.00	60.00

aBackground values of the study area; ^b^Dutch soil quality standard [56]; ^c^Canadian environmental quality guidelines [57]; ^d^Toxicity reference value [39]; ^e^ Australia soil quality standard [58].

Higher concentrations of Cd found in soil was 2.05 ± 0.84, 1.25 ± 0.86, 1.24 ± 0.56, and 1.21 ± 0.58 mg/kg at the MW, BS, WDA, and IA sites, correspondingly, which could be generated from the metal workshop, industrial activity and Cd plated substances [59]. For Cd concentration in the soil, about 80 % and 66 % of samples exceeded TRV, and DSWS, respectively (Table 4). The findings of this study were compared with previous studies presented in Table 5.

**Table 5:** Comparison of heavy metal concentration (mg/kg) (range) in soils of Jashore districts with other studies.

Study sites	As	Cd	Pb	Cr	Ni	Cu	References
Jashore (Bangladesh)	(1.80–18.09)	(0.10–3.58)	(0.40–113.26)	(0.90–72.09)	(2.10–68.23)	(3.82–212.57)	This study
Jhenaidah and Kushtia (Bangladesh)	(1.28–23.40)	(0.13–7.50)	(1.15–115.00)	(0.08–23.0)	(1.02–77.30)	(0.99–123.00)	[17]
Patuakhali (Bangladesh)	(0.26–80.0)	(0.17–24.0)	(9.40–276.00)	(1.30–74.00)	(4.90–27.00)	(4.10–122.00)	[45]
Dhaka (Bangladesh)	(8.7–277.00)	(1.8–80.00)	(13.00–706.00)	(2.40–1258.00)	(8.30–923.00)	(10–701)	[7], [9]
Linfen (China)	(8.08–32.56)	X	(15.50–143.32)	(21.02–101.10)	(15.30–60.57)	(2.02–66.35)	[60]
Medak province (India)	(0.4–14.00)	(0.1–4.20)	(5.00–77.00)	(81.00–751.00)	(1.00–50.00)	(2.00–180.00)	[61]
Shiraz (Iran)	(2.50–7.90)	(0.25–0.30)	(6.00–55.00)	(94.00–758.00)	(6.90–15.00)	(20.00–102.00)	[62]
Klang District (Malaysia)	X	(0.12–5.20)	(1.12–157.40)	(0.30–60.67)	X	(2.38–139.23)	[4]
Beni-Mellal (Morocco)	X	(1.56–2.70)	(24.79–37.48)	(90.50–256.22)	X	(23.93–52.75)	[63]

### 3.2 Geochemical indices for soil contamination assessment

*Contamination factor (CF), Enrichment factor (EF), Geo-accumulation index (I*_*geo*_*), and Pollution load index (PLI)*.

The CF values for As ranged from 1.05—2.32, indicating a "moderate contamination" (Table 6). The range of CF for Cd (2.01—6.84) displays a medium to high contamination. Pb (0.2—3.92) and Cu (0.46—4.62) were low to considerable contamination levels, whereas most of the sampling points for Cr and Ni suggested low contamination.

**Table 6:** Contamination factor, degree of contamination and contamination level of potentially toxic elements in soils collected from Jashore district, Bangladesh.

Land use type	Contamination factor (CF)						Degree of contamination (C_d_)	Contamination level
	As	Cd	Pb	Cr	Ni	Cu		
PA	1.74	2.83	0.34	0.95	0.59	0.58	7.03	Moderate
PG	1.61	2.58	0.43	0.74	0.71	0.62	6.69	Moderate
M	1.05	3.26	1.22	1.19	0.42	1.19	8.33	Moderate
BF	2.07	3.62	1.44	0.98	0.94	1.24	10.29	Considerable
IA	1.36	4.03	3.92	0.72	0.75	0.71	11.49	Considerable
PP	1.39	3.14	0.77	0.82	0.46	1.05	7.63	Moderate
MW	2.32	6.84	3.22	0.82	2.34	4.62	20.16	High
BS	1.42	4.16	1.39	1.00	0.65	1.56	10.18	Considerable
PS	1.58	3.14	0.47	0.92	0.78	0.97	7.86	Moderate
RA	1.15	2.01	0.20	0.14	0.46	0.46	4.42	Low
WDA	1.27	4.15	1.49	0.94	0.86	1.22	9.93	Moderate

Conversely, it was discovered that the total CF values for all PTEs at research sites decreased the value of Cd > As > Pb > Cu > Cr > Ni, representing that Cd and As potentially create a concern for the ecosystems in the area. Considering the *C*_*d*_, the decreasing order of metal contamination sites as MW > IA > BF > WDA > BS > M > PS > PP > PA > PG > RA (Table 6). In the study area, PA, PG, M, PP, PS, and WDA show moderate and considerable contamination levels indicated by BF, IA, and BS. On the other hand, RA and MW show low and high degrees of contamination, respectively, due to the continuous dumping of waste materials and the discharge of industrial and urban effluent (Islam et al., 2015).

The EF is a standardized technique that is frequently used to classify the metal concentrations related to soils [42], [15]. The highest value of EFs was found for Cd (24.74) and the lowest values for Cr (0.53) indicate that the studied metals were minimal to enrich soil significantly. The research locations' EFs were arranged in descending order as follows: MW > PP > WDA > BF > BS > PA > PS > M > PP > PG > PA (Table 7). For all sample sites combined, the EF of all PTEs was in the smallest to largest of Cd > As > Pb > Cu > Ni > Cr (Table 7). The study findings indicated that anthropogenic activities (EF>1.5) are mostly responsible for the origin of PTEs in the urban soil of the Jashore district, while this study also observed lower enrichment (EF<1.5) values that show metals in the soil come from crustal origin.

**Table 7:** Enrichment factor (EF) and Geoaccumulation index (Igeo) value of potentially toxic elements in soils collected from Jashore districts, Bangladesh.

Land use type	Enrichment factor (EF)						Geoaccumulation index (*I*_*geo*_)					
	As	Cd	Pb	Cr	Ni	Cu	As	Cd	Pb	Cr	Ni	Cu
PA	5.81 ± 2.94	10.07 ± 2.20	1.09 ± 0.98	3.39 ± 1.56	2.20 ± 1.34	2.05 ± 0.84	0.22	0.92	-2.15	-0.66	-1.34	-1.37
PG	4.07 ± 2.37	6.59 ± 3.54	1.08 ± 0.55	1.87 ± 0.66	1.84 ± 0.78	1.57 ± 0.56	0.11	0.78	-1.80	-1.01	-1.07	-1.27
M	2.77 ± 1.06	8.58 ± 3.68	3.34 ± 1.54	3.19 ± 1.16	1.15 ± 0.60	3.30 ± 1.20	-0.51	1.12	-0.29	-0.34	-1.83	-0.33
BF	5.75 ± 2.38	10.37 ± 6.95	3.79 ± 0.90	2.61 ± 0.81	2.51 ± 0.82	3.37 ± 0.93	0.47	1.27	-0.06	-0.61	-0.67	-0.27
IA	7.34 ± 6.18	17.81 ± 3.28	19.46 ± 11.79	3.70 ± 1.76	3.70 ± 2.11	3.62 ± 1.85	-0.14	1.42	1.39	-1.06	-1.00	-1.07
PP	3.71 ± 1.39	8.78 ± 3.44	2.14 ± 1.28	2.27 ± 0.82	1.23 ± 0.64	2.91 ± 1.55	-0.11	1.07	-0.95	-0.87	-1.72	-0.51
MW	8.17 ± 3.46	24.74 ± 14.25	11.23 ± 4.72	2.87 ± 1.22	8.48 ± 3.26	17.22 ± 10.03	0.63	2.19	1.10	-0.88	0.64	1.62
BS	3.20 ± 1.99	10.69 ± 8.79	3.39 ± 1.88	2.46 ± 1.19	1.67 ± 0.74	4.12 ± 2.99	-0.08	1.47	-0.10	-0.58	-1.21	0.05
PS	4.61 ± 2.46	9.05 ± 6.22	1.39 ± 0.92	2.70 ± 0.94	2.27 ± 0.95	2.82 ± 1.24	0.08	1.07	-1.66	-0.70	-0.95	-0.63
RA	4.26 ± 1.77	8.14 ± 5.87	0.79 ± 0.81	0.53 ± 0.45	1.73 ± 0.74	1.77 ± 1.04	-0.37	0.42	-2.87	-3.39	-1.70	-1.69
WDA	4.18 ± 1.48	14.00 ± 6.41	5.13 ± 2.20	3.30 ± 1.57	3.06 ± 1.76	4.25 ± 2.04	-0.23	1.47	-0.01	-0.67	-0.80	-0.30

The *I*_*geo*_ values were − 0.51—0.63, 0.42—2.19, − 2.87—1.39, − 3.39— (−0.34), − 1.83—0.64 and − 1.69—1.62 for As, Cd, Pb, Cr, Ni and Cu, individually. The study sites *I*_*geo*_ rankings in descending order were as follows: MW > BF > BS > IA > WDA > M > PS > PP > PG > PA > RA (Table 7).

For all sample sites combined, the *I*_*geo*_ of all PTEs were in descending order as follows: Cd > As > Cu > Pb > Cr > Ni (Table 7). The study area soils were moderate to extremely contaminated with Cd due to air emissions, battery leachates, and Cd-plated materials, while other toxic metals were uncontaminated. This outcome also matches with other studies [7], [9], [17], [45].

**Fig. 2:**
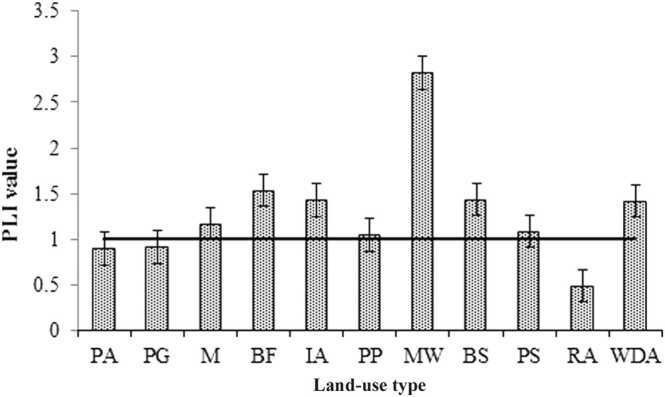
PLI values in the study area.

PLI is the most effective technique for evaluating the degree of toxic metal pollution. PLI values in this research ranged from 0.48–2.82, with an average of 1.29 (Fig. 2). The majority of the study site soils are contaminated (PLI > 1) by PTEs, indicating that their soil quality is deteriorating gradually. Site MW (2.82) has the highest PLI values, followed by BF (1.53) and IA (1.43) (Fig. 2) sites recommended that activities from the metal workshop, industries, and emissions from the brick field make the study area more harmful for surrounding ecosystems. The results of the overall evaluation showed that the investigated area is contaminated and faces serious threats to the surrounding environment. [17] found a significant level of PLI in their study area (Jhenaidah and Kushtia district, Bangladesh).

### 3.3 Assessment of potential ecological risk

The results of the PER valuation revealed that the *E*_*ri*_ value for every metal at each location was, in descending order: Cd > As > Pb > Cu > Ni > Cr (Table 8). Cadmium (Cd) among the metals analyzed, showed a moderate to high ecological risk, and with *E*_*ri*_ values varying from 60.25 to 205.13 (Table 8). Various anthropogenic factors, including the usage of phosphate fertilizers, the discharge of oily wastewater, and waste products from domestic and commercial operations, could be the cause of the presence of Cd in urban soil. The *E*_*ri*_ values for other studied toxic metals (As, Ni, Pb, Cu, and Cr) indicate minimal ecological risk because of their minor toxic response factor (*T*_*ri*_). On the other hand, PERI shows the sensitivity of numerous biological species to toxic elements and demonstrates the probable ecological hazard triggered by the toxic elements. Islam et al. [45] found that Cd contributes significantly to the PER in the soil of Patuakhali District, Bangladesh.

**Table 8:** Potential ecological risk factor, risk index and risk level of potentially toxic elements in soils collected from Jashore district, Bangladesh.

Land use type	Potential ecological risk factor (E^i^_r_)						Potential ecological risk (PER)	Risk level
	As	Cd	Pb	Cr	Ni	Cu		
PA	17.46	85.0	1.69	1.89	3.54	2.90	112.49	Moderate
PG	16.14	77.5	2.15	1.48	4.29	3.11	104.67	Moderate
M	10.52	97.75	6.12	2.38	2.53	5.95	125.25	Moderate
BF	20.74	108.63	7.22	1.96	5.64	6.20	150.39	Considerable
IA	13.64	120.75	19.59	1.44	4.50	3.56	163.49	Considerable
PP	13.90	94.25	3.87	1.64	2.74	5.27	121.68	Moderate
MW	23.24	205.13	16.09	1.63	14.05	23.09	283.22	Very high
BS	14.20	124.67	6.97	2.01	3.89	7.79	159.53	Considerable
PS	15.85	94.25	2.37	1.85	4.66	4.85	123.84	Moderate
RA	11.57	60.25	1.02	0.29	2.77	2.32	78.23	Moderate
WDA	12.80	124.40	7.44	1.88	5.18	6.09	157.78	Considerable

The PERI value for the diverse categories of land use follows the decrease in order of MW > IA > BS > WDA > BF > M > PS > PP > PA > PG > RA (Table 8). However, the PERI values for all types of land use are 78.23 − 283.22, showing considerable to very high ecological risk (Table 8), due to the continuous receiving of toxic metals from diverse sources. The highest value of PERI (283.22) was found in the MW site (Table 8) indicating a very high potential ecological risk for their surrounding biota.

### 3.4 Human health risk assessment

#### 3.4.1 Non-carcinogenic health risk assessment

**Fig. 3:**
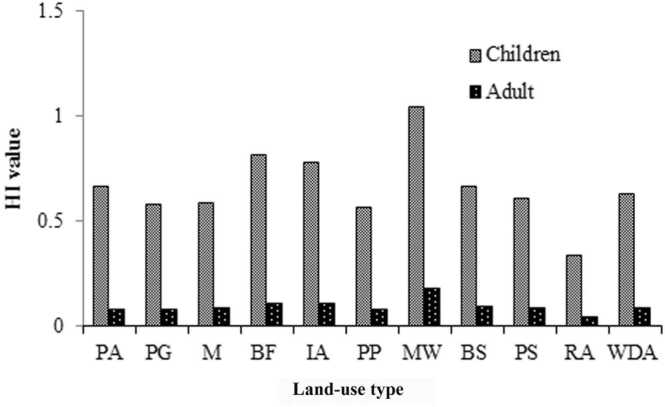
HI value for children and adults in the study area.

The potential non-carcinogenic health risk of individual mental for children and adults is determined by calculating HQ. The HQ value of each metal for ingestion, dermal contact, and inhalation routes are presented in Table S6, Table S7, and Table S8, respectively. Based on USEPA [48] guideline, HQ > 1, indicates non-cancer health risk is related to over-exposure. The study result shows that non-cancer health risks associated with each metal exposure via soil ingestion, dermal contact, and inhalation were lower than one (HQ < 1), suggesting a lower health risk for children and adults. The cumulative impacts of exposed metals were computed as HI (i.e., combining soil ingestion, dermatological contact, and inhalation). The HI values for children were 0.33–1.02 with an average of 0.65 ± 0.17 as well as for adults 0.04–0.17 with an average of 0.09 ± 0.03 (Fig. 3), except for one site (MW) for children, other all sampling sites for both children and adults indicating was a lower tendency of having non-cancer health risk.

The HI value for children was 7.22 times higher than adult inhabitants, demonstrating that children may pose 7.22 times greater chances of a non-cancer health risk than an adult in the future. Moreover, the THQ value for children was 7.82, 2.74, and 2.33 times higher than adults for ingestion, dermal contact, and inhalation exposure route, respectively. The findings of the current study conform to the observations of Ajeh et al. [64], and [17]. It demonstrates that children are more vulnerable to non-cancerous health risks of studied toxic metals than adults due to their high possibility of exposing contaminated particles through touching, mouthing, and playing behaviour [64]. Mainly, the oral ingestion route and concentration of As in soil are responsible for non-carcinogenic risks to children and adults. They might suffer from diverse non-carcinogenic health hazards including vomiting, skin diseases, respiratory tract irritation, cardiovascular, intestinal pain, hair loss, and neurological problems [16]. Ingestion was the prime route of exposing toxic elements in Benin City, Nigeria also found by Ajeh et al. [64].

#### 3.4.2 Carcinogenic health risk evaluation

The summary of the CRs evaluation is presented in Table 9. The decreasing order of different exposure routes for metals is ingestion > dermal contact > inhalation for both adults and children, indicating that ingestion is the most vital exposure route for creating potential cancer risk. The LTCR of studied heavy metal following descending order of As (2.10E-03) > Cd (6.69E-05) > Pb (3.25E-05) for children and As (2.74E-04) > Cd (3.17E-05) > Pb (4.33E-06) for adults (Table 9), suggesting that As is the most concerning metal for both children and adult which may pose adverse diverge health effects.

**Table 9:** Carcinogenic risk of children and adults due to ingestion, dermal contact, and inhalation of arsenic, cadmium and lead in soils collected from Jashore district, Bangladesh.

Target group	Route of exposure	As	Cd	Pb	Total risk
Children	Ingestion	1.60E-03	5.80E-05	3.24E-05	1.69E-03
	Dermal contact	5.00E-04	8.91E-06	9.95E-08	5.09E-04
	Inhalation	6.15E-08	2.23E-09	6.16E-09	6.99E-08
Adults	Ingestion	1.71E-04	6.21E-06	3.47E-06	1.81E-04
	Dermal contact	1.03E-04	2.55E-05	8.60E-07	1.30E-04
	Inhalation	2.64E-08	9.56E-10	2.64E-09	3.00E-08
Lifetime cancer risk (Children)		2.10E-03	6.69E-05	3.25E-05	2.20E-03
Lifetime cancer risk (Adults)		2.74E-04	3.17E-05	4.33E-06	3.10E-04

The overall cancer risk for adults and children was found to be 3.10E-04 and 2.20E-03, respectively, which were higher than recommended values (1E-06–1E-04) (Table 9). Table 9 also indicates that children have 7.10 times greater carcinogenic health risk than adults due to their lower body weight and higher exposure frequency. This study finding suggested that As is the main contributor to the overall cancer risks among the other studied heavy metals (Pb and Cd) in the urban soil of the Jashore district (Table 9). Though this study did not indicate any specific forms of arsenic-associated cancer in the study area. Several authors stated that long-term exposure to As might create different types of cancer such as skin, kidney, bladder, etc [16]. On the other hand, the chances of carcinogenic risk via oral exposure to metals-contaminated soil are high (Table 9), so to reduce health risks in the future, it is vital to minimize metal poisoning of urban soils in the Jashore district.

### 3.5 Principal component analysis (PCA), Pearson's correlation matrix (PCM), and cluster analysis (CA) for PTEs in soil samples

**Fig. 4:**
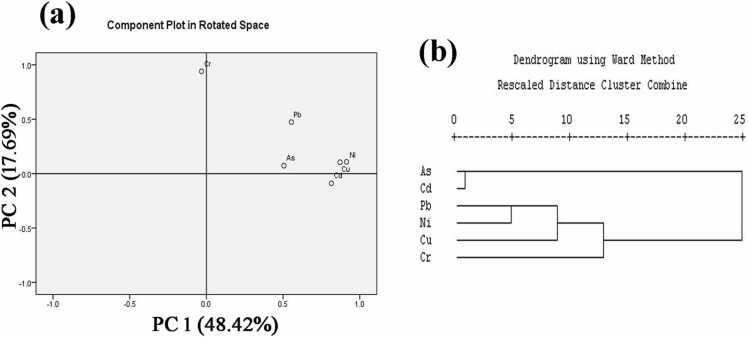
Multivariate analysis results [(a) PCA and (b) CA] for PTEs in soils collected from Jashore district, Bangladesh.

To understand the origin, dispersal, and mobility of the analyzed PTEs in the urban soil, multivariate investigations (PCM, CA, and PCA) are used (Fig. 4 and Table S9). In this study, PCA, CA, and PCM were used to find studied heavy metal sources precisely. Varimax rotation was used to perform PCA based on the concentrations of the tested PTEs. About 0.709 and 157.029 (df = 15, P = 0.000) were the values for Kaiser-Meyer-Olkin and Bartlett, respectively.

The PCA results revealed those two principal elements could account for 66.092 % of the total variation in the soil and could be used to represent the heterogeneity of PTEs. The primary principal component (PC1), which contributed 48.42 % of the total variance as well as loading with As, Cd, Pb, Ni, and Cu (Fig. 4a), which displays mutual linkage in the cluster diagram (Fig. 4b) with a considerable positive correlation [Table S9]. The contributors of As, Cd, Pb, Ni, and Cu in the study sites probably occur from road dust, the outdoor combustion of waste items, pollution from industry and automobiles, vehicle tires, excessive use of chemical fertilizers, farming practices and discharging of wastewater (e.g., rechargeable battery, metal workshops, metal plating, brake pads, arsenic sulfide, and cosmetic varnish for wood industries). The PC2 component reported 17.69 % of the overall variance. It displayed positive loading for Cr, which is separately positioned in the PCA, CA (Fig. 4a, b) and demonstrations no correlation between other metals (Table S9) signifying that the Cr had a separate lithogenic source. However multivariate analysis has some variations but it shows a good harmony for studied PTEs sources recognition. Lastly, the multivariate analysis outcome shows that manmade activities are governing rather than natural sources, which is a match with earlier findings of Bhuiyan et al., [65], Islam et al., [9], and Islam et al., [5].

### 3.6 The positive matrix factorization (PMF) approach for metal source attribution in soil

**Fig. 5:**
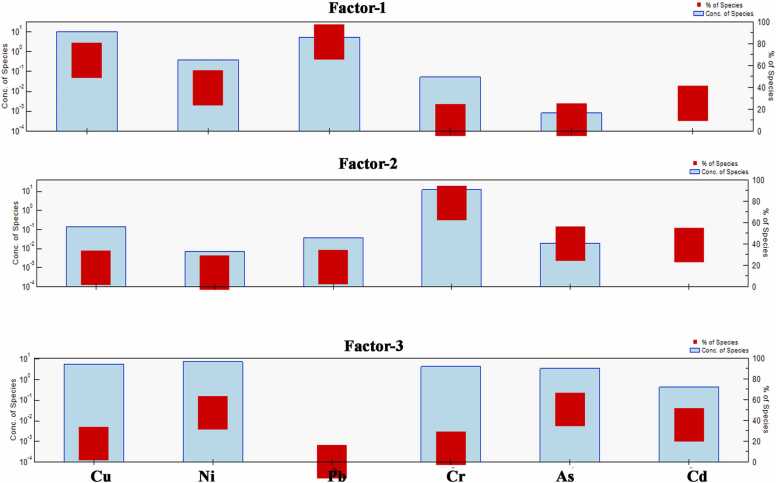
Profiles and contributions of sources of PTEs in soil samples from PMF model.

A common factorization receptor model, PMF (version 5.0), is used to determine each toxic metal's contribution as well as to identify and evaluate the likely sources of toxic metals in urban soil. In this study, Q was reduced to control the residual matrix and the residuals that were between − 1 and + 1. This was done to verify the model and determine its applicability. This system was run 20 times, with different parameters (3 or 5) being examined each time, to achieve a decent result. The association between experiential (R^2^) and tested value ranged from 0.82 to 0.99 indicating a strong relationship and 0.33 (Pb) is stated as the lower relationship for soil samples (Table S4). Therefore, the PMF model's distribution of the analyzed hazardous metals was accurate, and the outcomes were trustworthy. Factor 1 was vastly loaded with Pb (81.8 %) and Cu (64.7 %). Furthermore, factor 1 had a considerable amount of Ni (39.8 %), and Cd (25.7 %) (Fig. 5). Factor 1 was shown to be widely distributed throughout the research location. Lead was mainly coming from dust deposition from traffic emissions, vehicle tires, and brake pads [66]. While Cu in the study area comes from industrial activities (waste from metal and electrical working areas), waste burning, and sewage sludge [65], [67]. Hence, Factor 1 was considered a diverse anthropogenic source.

Factor 2 was dominated by Cr (78.1 %), As (40.2 %), and Cd (38.8 %) (Fig. 5). Chromium ranked 21st in the richness of elements inside the Earth's crust, causing exogenic geological processes (such as erosion and weathering) that contribute to the soils [68]. Besides, in the study area As and Cd was connected to urban and industrial pollutants, where waste from industrial activities, metal workshop, brickfield and burning coal and other fossil fuels, petrochemical might be a rational cause of As and Cd in the soils. Therefore, Cr is controlled by geogenic sources and As and Cd are anthropogenic inputs. Hence, factor 2 was attributable to diverse sources. Factor 3 was described by As (50.3 %), Ni (47.2 %), and Cd (35.5 %) (Fig. 5), where As and Cd were highly influenced by anthropogenic activities. Nickel in the soil may be resulting from the burning of sewage sludge and waste, as well as the combustion of coal, diesel, and fuel oil [69]. Finally, factor 3 is influenced by urban pollution.

## 4 Limitations of the study

This research provides important information regarding the presence of PTEs in the urban soil of Jashore, Bangladesh. In this study, seasonal variability was not taken into account. Additionally, PTEs in soil's bio-accessibility have not been determined, making it impossible to estimate actual exposure levels. Moreover, applying the risk assessment to these metrics makes the case even stronger. We defined the hazard quotient (HQ), hazard index (HI), and carcinogenic risk (CR) as the non-carcinogenic and carcinogenic risks of PTEs, respectively, where extra sources weren't taken into account. However, we examined 11 land use areas for six different toxic metals. Due to resource limitations, other metals and land-use sites could not be included in this study. These problems should be considered in future work.

## 5 Conclusions

Soil from the metal workshop, industrial area, and brick filed site were more heavily contaminated by studied PTEs than in other land-use areas, demonstrating that metal concentrations in the soils of the studied region varied with changes in land-use. Concentrations of Pb, As, Cr, Cu, Ni, and Cd in some sites exceeded background, soil quality indices, and soil quality standard values suggesting that soils were low to high polluted by PTEs. The substantial presence of toxic metals in soils was demonstrated by positive matrix factorization model fitting coefficients for PTEs, which exhibited R^2^ > 0.70 and demonstrated the validity of the model's output. Mainly, anthropogenic activities were considered to enrich PTEs in the soils. According to the possible risk indices, only Cd had a very high risk for ecology, which ranged from moderate to very high-risk levels in the study area. This study indicates lower non-cancer risks of each metal for children and adults by exposing contaminated soil via ingestion, dermal contact, and inhalation. Still, these target groups had significant carcinogenic risks. Children are more suspected than adults compared to health risk vulnerabilities. However, considering the oral ingestion of PTEs, the risk to human health will increase. Therefore, proper actions have to be taken to considerably improve the soil quality and decrease the health risks to the residents. Further study is required to identify the toxico-dynamics of PTEs in the soil profile for taking decisions on human health and environmental risk management of the study area.

## Ethics approval and consent to participant

This article does not contain any studies involving human participants performed by any authors. These studies evaluate the potentially toxic elements I urban soil and calculate the human health risk based on the assessed concentrations. No further toxicological experiments were conducted on any other living species. The manuscript in part or in full has not been submitted or published anywhere.

## Funding

This research did not receive any specific grant from funding agencies in the public, private, or not-for-profit sectors.

## CRediT authorship contribution statement

Tapos Kumar Chakraborty, Gopal Chandra Ghosh, Prianka Ghosh, and Samina Zaman: Conceived and designed the experiments; Md Ripon Hossain, Md. Shahnul Islam, Ahsan Habib, Sozibur Rahman, Khandakar Rashedul Islam, Baytune Nahar Netema and Md. Simoon Nice: Performed the experiments, and sample collection; Tapos Kumar Chakraborty, Nazmul Hossain, Suvasish Das Shuvo, Prianka Ghosh, and Samina Zaman: Analyzed and interpreted the data; Gopal Chandra Ghosh and Abu Shamim Khan: Contributed reagents, materials, analysis tools or data; Tapos Kumar Chakraborty, Prianka Ghosh, Md Shahnul Islam, Ahsan Habib and Samina Zaman: Wrote the paper.

## Declaration of Competing Interest

The authors declare that they have no known competing financial interests or personal relationships that could have appeared to influence the work reported in this paper.

## Data Availability

Data will be made available on request.
