# Supplementary material for: Receptor model-based sources and risks appraisal of potentially toxic elements in the urban soils of Bangladesh
Source: Toxicol Rep. 2023 Feb 24;10:308–19. doi: 10.1016/j.toxrep.2023.02.011 (PMC9986644; doi:10.1016/j.toxrep.2023.02.011)
Supplement: Supplementary file 1 — Supplementary material [file mmc1.docx]

**Supplementary information**

**Receptor model-based sources and risks appraisal of potentially toxic elements in the urban soils of Bangladesh**

Tapos Kumar Chakraborty^a*^, Md Shahnul Islam^a^, Gopal Chandra Ghosh^a^, Prianka Ghosh^a^, , Samina Zaman^a^, Md Ripon Hossain^a^, Ahsan Habib^a^, Md. Simoon Nice^a^, Md. Sozibur Rahman^a^, Khandakar Rashedul Islam^a^ , Baytune Nahar Netema^a^, Suvasish Das Shuvo^b^, Nazmul Hossain^c^ and Abu Shamim Khan^d^

^a^Department of Environmental Science and Technology, Jashore University of Science and Technology, Jashore 7408, Bangladesh.

^b^Department of Nutrition and Food Technology, Jashore University of Science and Technology, Jashore 7408, Bangladesh.

^c^Department of Computer Science and Engineering, Jashore University of Science and Technology, Jashore 7408, Bangladesh.

^d^Environmental Laboratory, Asia Arsenic Network, Jashore 7400, Bangladesh.

*Corresponding Author: E-mail: [taposchakraborty@just.edu.bd](mailto:taposchakraborty@just.edu.bd)

**Table S1.** Relationship between metals certified and recovery values (mean ± standard errors in mg/kg).

| Elements | Certified value | Measured value | Deviation (%) | Recovery (%) |
| --- | --- | --- | --- | --- |
| As | 10.00± 0.02 | 9.32±0.26 | 7.30 | 93.20 |
| Ni | 2.00±0.05 | 2.25±0.03 | 11.11 | 112.50 |
| Cd | 0.40±0.02 | 0.37±0.00 | 8.11 | 92.50 |
| Cr | 10.00±0.10 | 8.74±0.22 | 12.60 | 87.40 |
| Cu | 2.00±0.12 | 2.22±0.02 | 9.90 | 111.00 |
| Fe | 1.00±0.10 | 0.93±0.06 | 7.52 | 93.00 |
| Pb | 20.00±0.15 | 17.63±0.13 | 11.85 | 88.15 |

**Table S2.** Pollution / risk evaluation categories

| **Indices** | **Grade** | **Comments** | **References** |
| --- | --- | --- | --- |
| Enrichment factor (EF) | EF < 2 | Minimal enrichments | Sutherland (2020) |
|  | 2 ≤ EF < 5 | Moderate enrichments |  |
|  | 5 ≤ EF < 20 | Significantly enriched |  |
|  | 20 ≤ EF < 40 | Very highly enriched |  |
|  | EF ≥ 40 | Extremely enriched |  |
| Geo-accumulation index (*I_geo_*) | *I_geo_* ≤ 0 | Unpolluted | Muller (1979) |
|  | *I_geo_* = 0-1 | Unpolluted to moderately polluted |  |
|  | *I_geo_* = 1-2 | Moderately polluted |  |
|  | *I_geo_* = 2-3 | Moderately to strongly polluted |  |
|  | *I_geo_* = 3-4 | Strongly polluted |  |
|  | *I_geo_* = 4-5 | Strongly to extremely polluted |  |
|  | *I_geo_* > 5 | Extremely polluted |  |
| Contamination factor (*CF_i_*) | *CF* < 1 | Low | Hakanson (1980), Luo et al. (2007) |
|  | 1 ≤ *CF* < 3 | Moderate |  |
|  | 3 ≤ *CF* < 6 | Considerable |  |
|  | *CF* ≥ 6 | High |  |
| Degree of contamination (*C_d_*) | *C_d_* < 5 | Low | Hakanson (1980), Luo et al. (2007) |
|  | 5 ≤ *C_d_ <* 10 | Moderate |  |
|  | 10 ≤ *C_d_* < 20 | Considerable |  |
|  | *C_d_* ≥ 20 | High |  |
| Pollution load index (PLI) | *PLI* < 1 | No deterioration | Tomlinson et al. (1980) |
|  | *PLI* = 1 | Baseline |  |
|  | *PLI* > 1 | Progressive deterioration |  |
| Potential ecological risk evaluation index (*PERI*) | *E_r_^i^* < 40 | Low | Hakanson (1980) |
|  | 40 ≤ *E_r_^i^* < 80 | Moderate |  |
|  | 80 ≤ *E_r_^i^* < 160 | Considerable |  |
|  | 160 ≤ *E_r_^i^* < 320 | High |  |
|  | *E_r_^i^* ≥ 320 | Very high |  |
| Hazard quotient (*HQ*) | *HQ* < 1 | No significant non-carcinogenic health risk |  |
|  | *HQ* > 1 | Major non-carcinogenic health hazards |  |
| Hazard index (*HI*) | *HI* < 0.1 | Negligible | USEPA (1999) |
|  | 0.1 ≤ *HI* < 1 | Low |  |
|  | 1 ≤ *HI* < 4 | Medium |  |
|  | *HI* ≥ 4 | High |  |
| Carcinogenic risk (*CR*) | *CR* <1E-06 | Very low | USEPA (1999) |
|  | 1E-06 < *CR* < 1E-05 | Low |  |
|  | 1E-05 < *CR* < 1E-04 | Medium |  |
|  | 1E-04 < *CR* < 1E-03 | High |  |
|  | *CR* > 1E-03 | Very high |  |

**Table S3.** Reference doses (*RfD*) in (mg/kg/day) and Cancer Slope Factors (*CSF*) for the different heavy metals (Sources: Kamunda et al., 2016, Islam et al., 2020, Fan et al., 2021).

|  | *RfD* (mg/kg/day) | | | CSF (mg/kg/day) | | |
| --- | --- | --- | --- | --- | --- | --- |
| Toxic Metal | Ingest | Dermal | Inhale | Ingest | Dermal | Inhale |
| As | 3.00E-04 | 3.00E-04 | 1.50E-05 | 1.50E+00 | 3.66E+00 | 1.50E+00 |
| Ni | 2.00E-02 | 2.00E-02 | 9.00E-05 |  |  |  |
| Cd | 1.00E-03 | 1.00E-03 | 1.00E-05 | 3.80E-01 | 6.30E+00 | 3.80E-01 |
| Cr | 3.00E-03 | 3.00E-03 | 1.00E-04 |  |  |  |
| Cu | 3.70E-02 | 3.70E-02 | 5.00E-03 |  |  |  |
| Pb | 3.60E-03 | 3.60E-03 | 5.00E-04 | 8.50E-03 | 8.50E-03 | 4.20E-02 |

**Table S4.** PMF model accuracy data

| Elements | Category | R^2^ | Intercept | Intercept SE | Slope | Slope SE | SE | Normal Resided |
| --- | --- | --- | --- | --- | --- | --- | --- | --- |
| Cu | Strong | 0.97 | 4.008 | 2.125 | 0.820 | 0.047 | 4.531 | Yes |
| Ni | Strong | 0.99 | 2.001 | 0.554 | 0.873 | 0.028 | 1.203 | Yes |
| Pb | Strong | 0.33 | 8.111 | 7.403 | 0.454 | 0.212 | 16.294 | Yes |
| Cr | Strong | 0.98 | -0.285 | 1.756 | 1.006 | 0.048 | 1.708 | Yes |
| As | Strong | 0.90 | 0.019 | 0.813 | 0.988 | 0.109 | 0.700 | Yes |
| Cd | Strong | 0.83 | 0.052 | 0.190 | 0.932 | 0.143 | 0.205 | Yes |

**Table S5.** Physicochemical characteristics of soil (Mean ± SD) collected from Jashore districts, Bangladesh.

| Land use type | pH | EC (μS/cm) | OM (%) | OC (%) | Sand (%) | Silt (%) | Clay (%) | Soil Texture |
| --- | --- | --- | --- | --- | --- | --- | --- | --- |
| PA | 7.36±0.37 | 235.66±54.09 | 2.57±0.95 | 1.49±0.55 | 74.25 | 23.25 | 2.5 | Sandy Loam |
| PG | 7.67±0.46 | 948.25±263.05 | 1.98±0.91 | 1.15±0.53 | 74.5 | 21.5 | 4 | Sandy Loam |
| M | 7.69±0.45 | 1185.37±198.72 | 2.91±1.07 | 1.69±0.62 | 87.75 | 11.75 | 2.5 | Sandy Loam |
| BF | 7.58±0.57 | 2082.37±744.73 | 1.95±1.09 | 1.13±0.63 | 87.25 | 8.25 | 4.5 | Sandy Loam |
| IA | 7.47±0.38 | 3036.75±225.35 | 3.64±1.48 | 2.11±0.86 | 77 | 20 | 3 | Sandy Loam |
| PP | 8.15±0.34 | 683.12±311.15 | 4.43±2.09 | 2.57±1.21 | 83 | 16 | 1 | Sandy Loam |
| MW | 7.81±0.45 | 1226.25±154.38 | 3.90±2.12 | 2.26±1.23 | 61 | 23 | 16 | Sandy Loam |
| BS | 8.10±0.50 | 1680.00±427.96 | 4.91±3.36 | 2.85±1.95 | 79 | 20 | 1 | Sandy Loam |
| PS | 7.72±0.60 | 853.75±319.72 | 1.59±0.90 | 0.92±0.52 | 64 | 33 | 3 | Sandy Loam |
| RA | 8.06±0.37 | 523.37±293.05 | 1.09±0.62 | 0.63±0.36 | 71 | 27 | 2 | Sandy Loam |
| WDA | 7.50±0.67 | 1024.8±212.29 | 5.65±1.90 | 3.28±1.10 | 82 | 16 | 2 | Sandy Loam |

**Table S6.** HQ value of potentially toxic elements via ingestion of soils collected from Jashore district, Bangladesh.

| Land-use type | As | | Cd | | Pb | | Cr | | Ni | | Cu | |
| --- | --- | --- | --- | --- | --- | --- | --- | --- | --- | --- | --- | --- |
|  | children | adult | children | adult | children | adult | children | adult | children | adult | children | adult |
| PA | 3.65E-01 | 3.91E-02 | 1.09E-02 | 1.16E-03 | 2.48E-02 | 2.65E-03 | 1.66E-01 | 1.77E-02 | 8.30E-03 | 8.90E-04 | 5.00E-03 | 6.12E-03 |
| PG | 3.37E-01 | 3.61E-02 | 9.97E-03 | 1.07E-03 | 3.14E-02 | 3.37E-03 | 1.30E-01 | 1.39E-02 | 1.00E-02 | 1.08E-03 | 5.37E-03 | 6.57E-03 |
| M | 2.19E-01 | 2.35E-02 | 1.25E-02 | 1.34E-03 | 8.95E-02 | 9.59E-03 | 2.08E-01 | 2.23E-02 | 5.94E-03 | 6.36E-04 | 1.03E-02 | 1.26E-02 |
| BF | 4.33E-01 | 4.64E-02 | 1.39E-02 | 1.49E-03 | 1.05E-01 | 1.13E-02 | 1.71E-01 | 1.84E-02 | 1.32E-02 | 1.42E-03 | 1.07E-02 | 1.31E-02 |
| IA | 2.85E-01 | 3.05E-02 | 1.55E-02 | 1.66E-03 | 2.86E-01 | 3.07E-02 | 1.26E-01 | 1.35E-02 | 1.05E-02 | 1.13E-03 | 6.15E-03 | 7.53E-03 |
| PP | 2.90E-01 | 3.11E-02 | 1.20E-02 | 1.29E-03 | 5.65E-02 | 6.06E-03 | 1.44E-01 | 1.54E-02 | 6.42E-03 | 6.88E-04 | 9.10E-03 | 1.11E-02 |
| MW | 4.85E-01 | 5.20E-02 | 2.62E-02 | 2.81E-03 | 2.35E-01 | 2.52E-02 | 1.43E-01 | 1.53E-02 | 3.29E-02 | 3.53E-03 | 3.98E-02 | 4.88E-02 |
| BS | 2.97E-01 | 3.18E-02 | 1.60E-02 | 1.71E-03 | 1.02E-01 | 1.09E-02 | 1.75E-01 | 1.88E-02 | 9.12E-03 | 9.77E-04 | 1.34E-02 | 1.65E-02 |
| PS | 3.31E-01 | 3.55E-02 | 1.20E-02 | 1.29E-03 | 3.47E-02 | 3.71E-03 | 1.61E-01 | 1.73E-02 | 1.09E-02 | 1.17E-03 | 8.37E-03 | 1.02E-02 |
| RA | 2.42E-01 | 2.59E-02 | 7.67E-03 | 8.22E-04 | 1.49E-02 | 1.60E-03 | 2.50E-02 | 2.68E-03 | 6.50E-03 | 6.97E-04 | 4.01E-03 | 4.90E-03 |
| WDA | 2.67E-01 | 2.86E-02 | 1.59E-02 | 1.70E-03 | 1.09E-01 | 1.16E-02 | 1.64E-01 | 1.76E-02 | 1.21E-02 | 1.30E-03 | 1.05E-02 | 1.29E-02 |

**Table S7.** HQ value of potentially toxic elements via dermal contact of soils collected from Jashore district, Bangladesh.

| Land use type | As | | Cd | | Pb | | Cr | | Ni | | Cu | |
| --- | --- | --- | --- | --- | --- | --- | --- | --- | --- | --- | --- | --- |
|  | children | adult | children | adult | children | adult | children | adult | children | adult | children | adult |
| PA | 4.67E-02 | 9.68E-03 | 1.39E-03 | 2.88E-04 | 3.09E-03 | 6.39E-04 | 2.12E-02 | 4.39E-03 | 1.06E-03 | 2.20E-04 | 6.92E-04 | 1.43E-04 |
| PG | 4.32E-02 | 8.95E-03 | 1.60E-06 | 2.65E-04 | 4.89E-06 | 8.10E-04 | 2.08E-05 | 3.44E-03 | 1.61E-06 | 2.67E-04 | 9.28E-07 | 1.54E-04 |
| M | 2.81E-02 | 5.83E-03 | 2.00E-06 | 3.32E-04 | 1.39E-05 | 2.31E-03 | 3.32E-05 | 5.51E-03 | 9.50E-07 | 1.58E-04 | 1.78E-06 | 2.95E-04 |
| BF | 5.55E-02 | 1.15E-02 | 2.23E-06 | 3.70E-04 | 1.64E-05 | 2.72E-03 | 2.74E-05 | 4.55E-03 | 2.12E-06 | 3.51E-04 | 1.85E-06 | 3.07E-04 |
| IA | 3.65E-02 | 7.56E-03 | 2.48E-06 | 4.11E-04 | 4.45E-05 | 7.39E-03 | 2.01E-05 | 3.34E-03 | 1.69E-06 | 2.80E-04 | 1.06E-06 | 1.77E-04 |
| PP | 3.72E-02 | 7.70E-03 | 1.92E-06 | 3.19E-04 | 8.80E-06 | 1.46E-03 | 2.30E-05 | 3.81E-03 | 1.03E-06 | 1.70E-04 | 1.57E-06 | 2.61E-04 |
| MW | 6.22E-02 | 1.29E-02 | 4.19E-06 | 6.95E-04 | 3.66E-05 | 6.07E-03 | 2.28E-05 | 3.78E-03 | 5.27E-06 | 8.74E-04 | 6.89E-06 | 1.14E-03 |
| BS | 3.80E-02 | 7.87E-03 | 2.56E-06 | 4.24E-04 | 1.59E-05 | 2.63E-03 | 2.81E-05 | 4.65E-03 | 1.46E-06 | 2.42E-04 | 2.32E-06 | 3.85E-04 |
| PS | 4.24E-02 | 8.79E-03 | 1.92E-06 | 3.19E-04 | 5.39E-06 | 8.94E-04 | 2.58E-05 | 4.28E-03 | 1.75E-06 | 2.90E-04 | 1.45E-06 | 2.40E-04 |
| RA | 3.10E-02 | 6.41E-03 | 1.23E-06 | 2.04E-04 | 2.32E-06 | 3.85E-04 | 4.00E-06 | 6.64E-04 | 1.04E-06 | 1.73E-04 | 6.93E-07 | 1.15E-04 |
| WDA | 3.42E-02 | 7.09E-03 | 2.54E-06 | 4.21E-04 | 1.69E-05 | 2.80E-03 | 2.63E-05 | 4.36E-03 | 1.94E-06 | 3.22E-04 | 1.82E-06 | 3.01E-04 |

**Table S8.** HQ value of potentially toxic elements via inhalation of soils collected from Jashore district, Bangladesh.

| Land use type | As | | Cd | | Pb | | Cr | | Ni | | Cu | |
| --- | --- | --- | --- | --- | --- | --- | --- | --- | --- | --- | --- | --- |
|  | children | adult | children | adult | children | adult | children | adult | children | adult | children | adult |
| PA | 2.81E-04 | 1.20E-04 | 4.18E-05 | 1.79E-05 | 6.67E-06 | 2.86E-06 | 1.91E-04 | 8.19E-05 | 7.10E-05 | 3.04E-05 | 1.54E-06 | 8.91E-08 |
| PG | 2.59E-04 | 1.11E-04 | 3.84E-05 | 1.64E-05 | 8.46E-06 | 3.63E-06 | 1.50E-04 | 6.42E-05 | 8.59E-05 | 3.68E-05 | 1.65E-06 | 9.57E-08 |
| M | 1.69E-04 | 7.24E-05 | 4.82E-05 | 2.07E-05 | 2.41E-05 | 1.03E-05 | 2.40E-04 | 1.03E-04 | 5.08E-05 | 2.18E-05 | 3.16E-06 | 1.83E-07 |
| BF | 3.33E-04 | 1.43E-04 | 5.36E-05 | 2.30E-05 | 2.84E-05 | 1.22E-05 | 1.98E-04 | 8.48E-05 | 1.13E-04 | 4.84E-05 | 3.29E-06 | 1.91E-07 |
| IA | 2.19E-04 | 9.40E-05 | 5.95E-05 | 2.55E-05 | 7.71E-05 | 3.30E-05 | 1.45E-04 | 6.22E-05 | 9.02E-05 | 3.87E-05 | 1.89E-06 | 1.10E-07 |
| PP | 2.23E-04 | 9.57E-05 | 4.62E-05 | 1.98E-05 | 1.52E-05 | 6.53E-06 | 1.66E-04 | 7.10E-05 | 5.49E-05 | 2.35E-05 | 2.80E-06 | 1.62E-07 |
| MW | 3.73E-04 | 1.60E-04 | 1.01E-04 | 4.32E-05 | 6.33E-05 | 2.71E-05 | 1.65E-04 | 7.05E-05 | 2.82E-04 | 1.21E-04 | 1.23E-05 | 7.10E-07 |
| BS | 2.28E-04 | 9.78E-05 | 6.15E-05 | 2.64E-05 | 2.74E-05 | 1.18E-05 | 2.02E-04 | 8.68E-05 | 7.80E-05 | 3.34E-05 | 4.14E-06 | 2.40E-07 |
| PS | 2.55E-04 | 1.09E-04 | 4.62E-05 | 1.98E-05 | 9.33E-06 | 4.00E-06 | 1.86E-04 | 7.99E-05 | 9.34E-05 | 4.00E-05 | 2.58E-06 | 1.49E-07 |
| RA | 1.86E-04 | 7.97E-05 | 2.95E-05 | 1.27E-05 | 4.02E-06 | 1.72E-06 | 2.89E-05 | 1.24E-05 | 5.56E-05 | 2.38E-05 | 1.23E-06 | 7.14E-08 |
| WDA | 2.06E-04 | 8.81E-05 | 6.10E-05 | 2.61E-05 | 2.93E-05 | 1.25E-05 | 1.90E-04 | 8.13E-05 | 1.04E-04 | 4.45E-05 | 3.23E-06 | 1.87E-07 |

**Table S9.** Correlation coefficient matrix for potentially toxic elements in soils collected from Jashore district, Bangladesh.

|  | As | Cd | Pb | Cr | Ni | Cu |
| --- | --- | --- | --- | --- | --- | --- |
| As | 1 |  |  |  |  |  |
| Cd | 0.337** | 1 |  |  |  |  |
| Pb | 0.182 | 0.317** | 1 |  |  |  |
| Cr | 0.086 | -0.005 | 0.209 | 1 |  |  |
| Ni | 0.407** | 0.612** | 0.506** | 0.071 | 1 |  |
| Cu | 0.233 | 0.624** | 0.442** | 0.097 | 0.831** | 1 |

**. Correlation is significant at the 0.01 level (1-tailed).

*. Correlation is significant at the 0.05 level (1-tailed
